# Quantifying Fluorescence Lifetime Responsiveness of Environment-Sensitive Probes for Membrane Fluidity Measurements

**DOI:** 10.1021/acs.jpcb.3c07006

**Published:** 2024-02-28

**Authors:** Franziska Ragaller, Ellen Sjule, Yagmur Balim Urem, Jan Schlegel, Rojbin El, Dunja Urbancic, Iztok Urbancic, Hans Blom, Erdinc Sezgin

**Affiliations:** † Department of Women’s and Children’s Health, Science for Life Laboratory, Karolinska Institutet, 17165 Solna, Sweden; ‡ Weatherall Institute of Molecular Medicine, University of Oxford, OX39DS Oxford, United Kingdom; § Faculty of Pharmacy, University of Ljubljana, 1000 Ljubljana, Slovenia; ∥ Laboratory of Biophysics, Condensed Matter Physics Department, Jožef Stefan Institute, 1000 Ljubljana, Slovenia; ⊥ Science for Life Laboratory, Department of Applied Physics, Royal Institute of Technology, 17165 Solna, Sweden

## Abstract

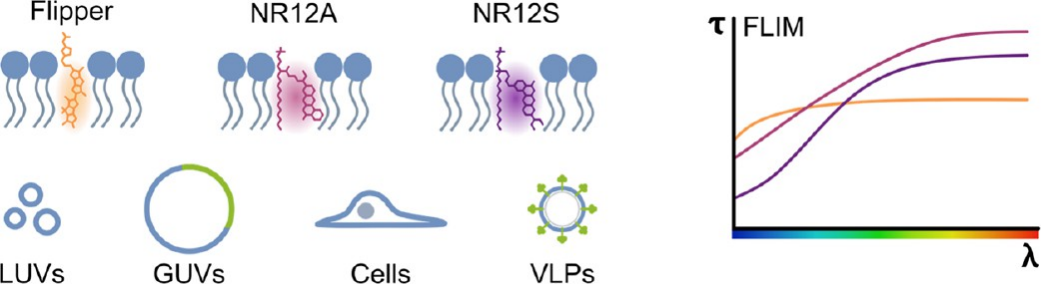

The structural diversity of different lipid species within the membrane defines its biophysical properties such as membrane fluidity, phase transition, curvature, charge distribution, and tension. Environment-sensitive probes, which change their spectral properties in response to their surrounding milieu, have greatly contributed to our understanding of such biophysical properties. To realize the full potential of these probes and avoid misinterpretation of their spectral responses, a detailed investigation of their fluorescence characteristics in different environments is necessary. Here, we examined the fluorescence lifetime of two newly developed membrane order probes, NR12S and NR12A, in response to alterations in their environments such as the degree of lipid saturation, cholesterol content, double bond position and configuration, and phospholipid headgroup. As a comparison, we investigated the lifetime sensitivity of the membrane tension probe Flipper in these environments. Applying fluorescence lifetime imaging microscopy (FLIM) in both model membranes and biological membranes, all probes distinguished membrane phases by lifetime but exhibited different lifetime sensitivities to varying membrane biophysical properties (e.g., cholesterol). While the lifetime of Flipper is particularly sensitive to the membrane cholesterol content, the NR12S and NR12A lifetimes are moderately sensitive to both the cholesterol content and lipid acyl chains. Moreover, all of the probes exhibit longer lifetimes at longer emission wavelengths in membranes of any complexity. This emission wavelength dependency results in varying lifetime resolutions at different spectral regions, which are highly relevant for FLIM data acquisition. Our data provide valuable insights on how to perform FLIM with these probes and highlight both their potential and limitations.

## Introduction

The plasma membrane is a highly complex organelle comprising different lipid species, various membrane-associated proteins, and glycocalyx components.^1^ This complexity ensures proper functionality of cellular mechanisms associated with the membrane such as cell signaling, intracellular membrane trafficking, endo- and exocytosis, and cell division.^2^ The asymmetric distribution of the more than hundred different lipid species of diverse structures within the membrane bilayer is also essential for these cellular processes.^3,4^ Determined by high structural diversity, the collective membrane biophysical properties, membrane fluidity, phase transition, curvature, charge distribution, and tension,^5,6^ vary dynamically as a result of alterations in local membrane composition.^7^ Membrane biophysical properties and cellular processes are closely intertwined, emphasizing the need for further in-depth investigation of biophysical properties.^6,8^

Investigating the influence of single structural changes (e.g., monounsaturated vs saturated lipids) on biophysical properties in a cell plasma membrane poses a challenge due to the high complexity of the plasma membrane. To circumvent this problem, model membrane systems allowing for custom lipid composition are often exploited.^1,9,10^ To examine membrane biophysical properties such as polarity, hydration, viscosity, and tension, among others, a variety of fluorescent probes have been developed in the past two decades, which respond to their associated environment by changes in intensity, emission wavelength, or fluorescence lifetime.^11,12^ Solvatochromic membrane probes distinguish between membranes comprising saturated vs unsaturated lipids by a shift of the emission maximum (toward longer wavelengths for membranes rich with unsaturated lipids).^11^ The classical solvatochromic probe Laurdan^13,14^ as well as other more recent probes such as di-4-ANEPPDHQ,^15^ NR12S,^16^ NR12A,^17^ NR4A,^17,18^ and Pro12A^19^ have proven very useful for cell biology.^20^ Especially, NR12S, NR12A, and Pro12A are advantageous compared to previously synthesized probes due to increased brightness, larger emission shift upon changes in the environment, a better defined plasma membrane location, and decreased internalization and cytotoxicity as well as their suitability for live-cell imaging.^16,17,19,20^ Their spectral shift is examined using spectral or ratiometric imaging and subsequently quantified by calculation of the generalized polarization parameter (GP) or intensity ratios using spectral fitting^21,22^ or fit-free spectral phasor approaches.^23^ Large red shifts result in low GP values and correlate with an increase in membrane fluidity, i.e., reporting on lower lipid order and decreased lipid packing.^14,21^ Of note, GP values or other ratios do not directly disclose the underlying biophysical mechanisms causing the emission shifts. Further, a common misconception is that all solvatochromic probes sense the same membrane biophysical properties, as recent data show that membrane probes Laurdan and di-4-ANEPPDHQ report on different membrane biophysical properties.^24^ Moreover, Pro12A, NR12S, and NR12A exhibit varying sensitivities toward lipid saturation index, cholesterol content, and configuration and position of lipid unsaturation and the lipid headgroup, which is due to varying locations and orientations of the probes in the membrane, resulting in subtle changes of their immediate environment.^25^ Finally, application of different techniques to measure the membrane order can lead to contradicting results, highlighting the need for direct comparative studies.^26^

Orthogonal to solvatochromic dyes, mechanosensitive planarizable push–pull probes (commercialized as Flipper-TR and referred to as “Flipper” hereon) that incorporate into membranes have been developed.^27−29^ These probes report on their environment by changing their fluorescence lifetime.^27^ The lifetime is examined using fluorescence lifetime imaging microscopy (FLIM) and subsequently analyzed by curve fitting, deconvolution, or phasor lifetime methods.^30^ Mechanistically, Flipper probes are planarized by increasing physical compression (membrane tension), thereby generating stronger push–pull systems, resulting in longer lifetimes.^27^ Flipper also senses increasing membrane order by an increase in lifetime and distinguishes liquid-ordered (Lo) and liquid-disordered (Ld) membrane phases.^28^ Other solvatochromic probes such as Laurdan,^31^ Nile Red,^32^ and di-4-ANEPPDHQ^33^ among others also report on their environment by changes in their lifetime. Di-4-ANEPPDHQ lifetime was shown to have better contrast for membrane phases than its spectral shift^33^ and was, for example, applied to investigate membrane asymmetry.^4^ Due to the intensity-independent nature, fluorescence lifetime measurements have advantages over intensity-based spectral measurements. Therefore, here, we aimed at investigating if the lifetimes of NR12S and NR12A, as an intensity-independent readout, can be reliably used as a parameter to quantify changes in biophysical properties of the membrane. Following extensive characterization of NR12S and NR12A using spectral imaging,^25^ we thus quantitatively examined if there are differences in sensitivity between the intensity-dependent emission shift and intensity-independent fluorescence lifetime. Furthermore, as a comparison, the sensitivity of Flipper as a lifetime probe to varying membrane biophysical properties was investigated. We determined the probes’ lifetimes in membranes of low complexity (large unilamellar vesicles (LUVs) and phase-separated giant unilamellar vesicles (GUVs)) and high complexity (cells and virus-like particles (VLPs)).

Overall, our results reveal that fluorescence lifetimes of NR12S and NR12A are mostly sensitive to a high cholesterol content and distinguish membrane phases. The lifetime of Flipper is particularly sensitive to the membrane cholesterol content, which may be related to an increase in membrane tension, and senses phase separation. Strikingly, all probes, especially NR12S and NR12A, exhibit longer lifetimes at longer emission wavelengths in membranes of any complexity. This emission wavelength dependency of the fluorescence lifetimes is important to consider when performing FLIM experiments with these probes, i.e., for the selection of detection parameters. To conclude, our data provide valuable insights on how to perform FLIM with these probes, in model membranes as well as more complex systems, highlighting both their potentials and limitations to explore membrane biophysical properties.

## Material and Methods

### Materials

The following lipids and environment-sensitive probes were utilized: 1,2-diarachidonoyl-*sn*-glycero-3-phosphocholine (DAPC, 20:4/20:4 PC), 1,2-dipetroselenoyl-*sn*-glycero-3-phosphocholine (Δ6cis DOPC, 18:1/18:1 PC), 1,2-dioleoyl-*sn*-glycero-3-phosphocholine (Δ9cis DOPC, 18:1/18:1 PC), 1,2-dielaidoyl-*sn*-glycero-3-phosphocholine (Δ9trans DOPC, 18:1/18:1 PC), 1-palmitoyl-2-oleoyl-glycero-3-phosphocholine (POPC, 16:0–18:1 PC), 1,2-dipalmitoyl-*sn*-glycero-3-phosphocholine (DPPC, 16:0/16:0 PC), 1-palmitoyl-2-oleoyl-*sn*-glycero-3-phospho-l-serine (POPS, 16:0/18:1 PS), 1-palmitoyl-2-oleoyl-*sn*-glycero-3-phosphoethanolamine (POPE, 16:0/18:1 PE), cholesterol, brain octadecanoyl sphingomyelin (SM, 18:0) (Avanti Polar Lipids), Flipper-TR,^28^ MemGlow NR12S,^16^ and NR12A^17^ membrane polarity probes. NaCl and HEPES were obtained from Sigma-Aldrich (St. Louis, MO). PBS, high-glucose DMEM, and Leibovitz’s L15 medium were acquired from ThermoFisher Scientific.

### Large Unilamellar Vesicle Preparation and Staining

For LUV production, the desired lipid mixture was prepared in chloroform (0.5 mg/mL). After removal of the solvent by a nitrogen flow, the lipid film was hydrated with 1 mL of buffer (150 mM NaCl, 10 mM HEPES, pH 7.4), and the solution was vortexed vigorously to disperse the lipid into the buffer. The solution was sonicated using a tip sonicator (power 3, duty cycle 40%) for 10 min. LUVs were stored under nitrogen at 4 °C. The LUVs were stained with 1 μM Flipper, NR12S, or NR12A. As a control (no environment-sensitive dye), 1 μM AlexaFluor 488 in water was used. All bulk samples were imaged in μ-Slides (18-well glass bottom, ibidi), previously blocked with 3 mg/mL BSA in PBS, at room temperature.

### Giant Unilamellar Vesicle Preparation and Staining

Phase-separated GUVs (SM:DOPC:Chol 2:2:1) were prepared according to a previously described protocol.^9^ Using custom-built GUV Teflon chambers with two platinum electrodes, GUVs were generated by electroformation.^34^ A volume of 6 μL of lipid dissolved in chloroform (1 mg/mL total lipid concentration) was homogeneously distributed on the electrodes, dried under a nitrogen stream, and placed in 300 nM sucrose solution (370 μL). Electroformation was performed at 2 V and 10 Hz at 70 °C (above the specific lipid transition temperature) for 1 h followed by 2 V and 2 Hz for 30 min. GUVs were stained at a final concentration of 300 nM Flipper and 100 nM NR12S or NR12A. The GUVs were imaged in μ-Slides (18-well glass bottom, ibidi), previously blocked with 3 mg/mL BSA in PBS, at room temperature.

### Cell Maintenance and Staining

NRK-52E, U-2 OS, RBL-2H3, and HEK293T cells were cultured in DMEM (high glucose, without pyruvate) with 10% FBS at 37 °C and 5% CO_2_. HEK293T cells were utilized for VLP preparation. For imaging, 1 × 10^4^ cells (NRK 52E, U2OS and RBL) per well were seeded in μ-Slides (18-well glass bottom, ibidi). Cells were stained with Flipper, NR12S, and NR12A (1 μM) in phenol red- and serum-free L15 medium. The cells were not washed before imaging.

### Preparation of Virus-Like Particles and Staining

VLPs were produced as described previously^35^ with small modifications. To produce pseudotyped nonfluorescent VLPs, HEK293T cells were seeded at a confluency of ∼70% in T75 flasks and cotransfected 6 h later with 7.5 μg of the lentiviral packaging vector psPAX2 (gift from Didier Trono, Addgene plasmid no. 12260) and 15 μg of the plasmid encoding the respective viral surface protein (pCMV14-3X-Flag-SARS-CoV-2 S was a gift from Zhaohui Qian, Addgene plasmid no. 145780; delta-spike expression plasmid kindly provided by Benjamin Murrell; Ebola GP expression plasmid kindly provided by Jochen Bodem) using Lipofectamine 3000 (ThermoFisher) according to the manufacturer’s recommendations. After 12 h, the medium was replaced by phenol red-free DMEM supplemented with 10% FCS, and VLPs were harvested twice after 24 h. The VLP-containing supernatant was sterile-filtered through a 0.45 μm PES filter and 50-fold enriched using a LentiX concentrator following the manufacturer’s protocol (Takara). The VLPs were diluted 1:1 with PBS and stained at a final concentration of 300 nM Flipper or 100 nM NR12S or NR12A before imaging in μ-Slides (18-well glass bottom, ibidi), previously blocked with 3 mg/mL BSA in PBS at room temperature. Of note, possible contamination by other particles of similar size and density such as lipoproteins or extracellular vesicles (EVs) cannot be excluded with the VLP preparation protocol utilized, possibly influencing the lifetime measurements by masking potential differences between VLP species.

### Normalized Excitation Spectra

The excitation spectra of Flipper, NR12S, and NR12A in Δ9*cis*DOPC LUVs were obtained using a Leica SP8 3X STED microscope, utilizing a STED white HC PL APO CS2 100*x*/1.40 oil objective. For Flipper, the excitation spectrum was measured from 470 to 570 nm (detection from 610 to 750 nm), and for NR12S and NR12A, it was measured from 470 to 630 nm (detection from 650 to 750 nm) in intervals of 2 nm. All excitation spectra were corrected for the background signal measured in pure buffer (150 mM NaCl, 10 mM HEPES, pH 7.4) and normalized to the maximum.

### Fluorescence Lifetime Imaging Microscopy

All FLIM measurements were performed on a Leica SP8 3X STED with a FALCON FLIM/FCS, utilizing a STED white HC PL APO CS2 100*x*/1.40 oil objective. Excitation output power was set to 70% of a pulsed white-light laser (pulse duration: ∼ 100–150 ps) and software-tuned to optimal settings for each experiment. All probes were excited at 488 nm, and emission was collected within the 500–700 nm range through prism-based spectral selections either in intervals of 20 nm (sequentially) for LUVs and VLPs or within 500–600 and 600–700 nm for phase-separated GUVs and cells. Emission was collected using an SMD HyD detector set to photon counting mode (10% internal gain). Power settings for FLIM ensured a maximum count rate of 0.5 photons per laser pulse, and all measurements were taken at 20 MHz repetition rate, except when investigating laser frequency influences where measurements at 40 and 80 MHz were performed. For more detailed information on specific acquisition parameters, see Table S1.

### Fluorescence Lifetime Analysis

**Figure 1:**
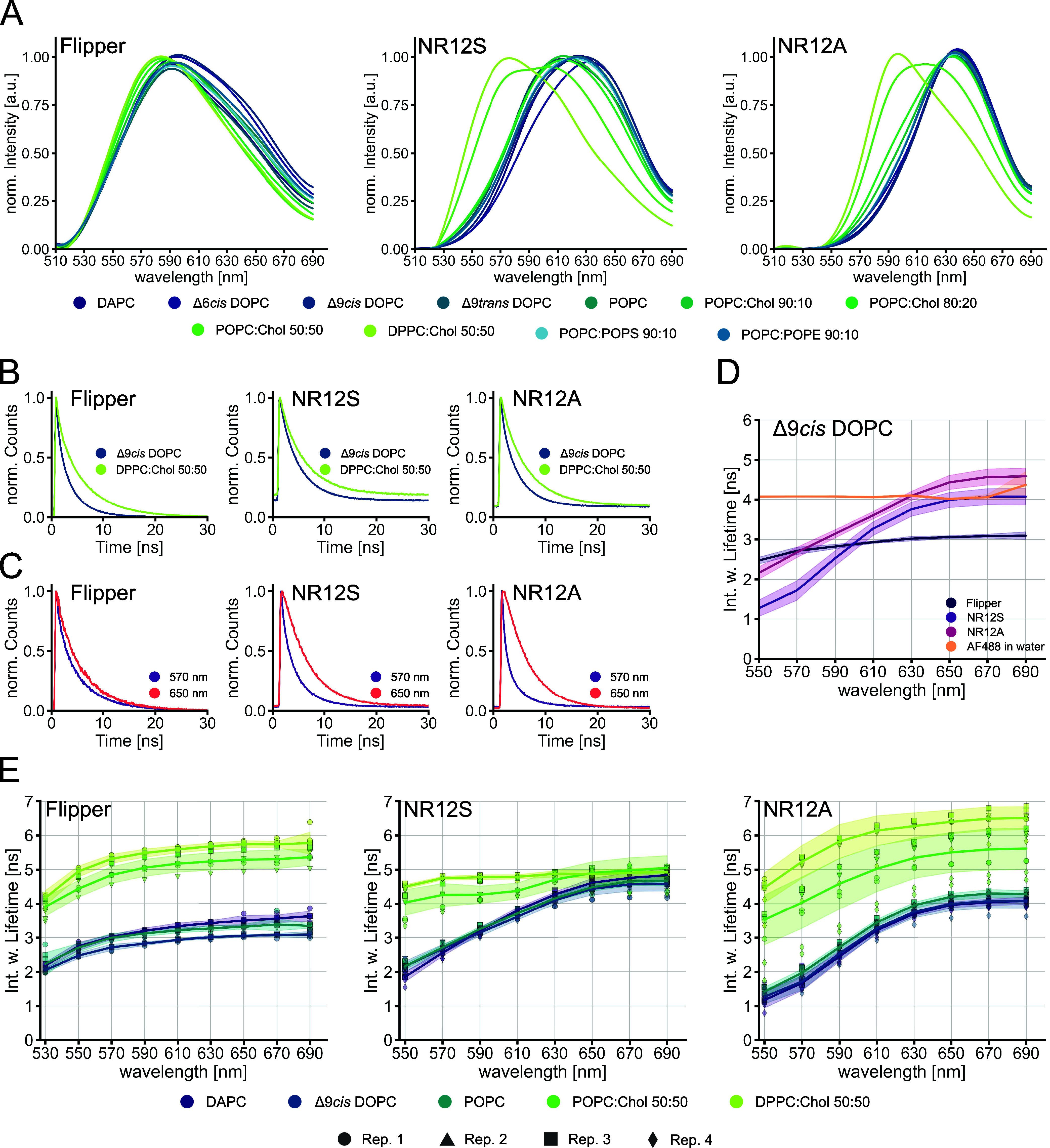
Characterization of Flipper, NR12S, and NR12A lifetimes in different lipid environments. Spectral fluorescence lifetime measurements of the probes in LUVs were carried out within 500–700 nm in intervals of 20 nm. Multiexponential curve fitting was performed for the fluorescence decays. (A) Normalized intensity spectra of Flipper (left), NR12S (middle), and NR12A (right) in varying lipid environments. (B) Normalized fluorescence decays (full spectrum, 500–700 nm) of Flipper (left), NR12S (middle), and NR12A (right) in Δ9*cis* DOPC or DPPC:Chol 50:50. (C) Normalized fluorescence decays at 570 nm vs 650 nm of Flipper (left), NR12S (middle), and NR12A (right) in POPC:Chol 80:20. (D) Spectrally resolved intensity-weighted lifetime of Flipper, NR12S, and NR12A in Δ9cis DOPC and the control AF488 in water. The line corresponds to the median of individual biological replicates (*n* ≥ 3). The band corresponds to the standard deviation. (E) Spectrally resolved intensity-weighted lifetime of Flipper (left), NR12S (middle), and NR12A (right) in different lipid environments with varying saturation indices. The line corresponds to the median of individual biological replicates shown with different symbols (*n* ≥ 3). The band corresponds to the standard deviation.

Lifetime analysis was performed by using Leica Application Suite LAS X FLIM/FCS software (version 4.5.0). All fluorescence decay curves were analyzed by n-exponential reconvolution fitting using the instrument response function (IRF) calculated by FALCON FLIM software within the range of 0.2–45 ns (20 MHz), 0.2–25 ns (40 MHz), and 0.2–12.5 ns (80 MHz). For all analyses, we further used an intrinsic standard (high-speed) photon filter, ensuring that only single photons detected between two laser pulses are used for the lifetime decay.^36^ For LUVs, cell and VLP whole-image analysis was performed (histograms of photons pooled from the whole image were fitted), whereas for phase-separated GUVs, histograms of photons from manually selected field of views were fitted (Figure S5). Different selected emission windows (i.e., width of 20 or 100 nm) were fitted individually, except for comparison of the decay rates of the probes in Δ9cis DOPC vs DPPC:Chol 50:50 LUVs (Figure 1B), where the decays across detection windows were combined (200 nm emission window). For the comparison of intensity-based vs lifetime-based analysis (Figure 5), the decays across detection windows within the range of 500–600 nm or 500–700 nm were combined, to ensure best lifetime resolution (according to Figures 1E and S2). Fluorescence decays of less than 10^4^ photons were excluded due to unreliable fitting analysis.^30,37^ Technical replicates exhibiting unrealistic lifetime values or inappropriate fits were identified as outliers and were excluded from the plots. For more detailed information on specific fitting parameters, see Table S2, especially for the selections of components used for n-exponential reconvolution fitting. All lifetime values shown in the plots correspond to the “mean intensity-weighted lifetime” calculated by LAS X FLIM/FCS software using eq 1
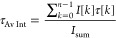
1with τ_Av Int_ being the ‘mean intensity-weighted lifetime’, *I* being the intensities associated with each exponential component, normalized to the time resolution of the measured decay curve, τ being the lifetime, and *I*_sum_ being the sum of fluorescence intensity for all components.

### Calculation of Normalized Intensity Spectra and GP

After determination of the lifetime values, all further analysis was carried out using Python 3.10. To calculate normalized intensity spectra, the sum intensity (*I*_sum_) obtained from the FLIM measurements was normalized to their maximum values. This was performed for each 20 nm emission window recorded for all probes across different lipid compositions. For better visualization, the normalized intensity values were interpolated using a cubic spline function.

To compare the resolution of lifetime and intensity of the probes in different lipid environments, the generalized polarization (GP) parameter was calculated using eq 2
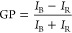
2with *I*_B_ and *I*_R_ being the fluorescence signal intensities at blue- and red-shifted emission wavelengths, respectively, for liquid-ordered, λ_Lo_, and liquid-disordered phases, λ_Ld_. The GP can adopt values ranging between +1 and −1, according to eq 1. The GP value is a ratiometric (relative) quantification depending on selected λ_Lo_ and λ_Ld_ values as well as the environment-sensitive probe. The respective sum intensity counts obtained from the FLIM measurements were used to calculate the median GP value (three replicates) of the investigated probes in different lipid environments. The median GP values were then compared with the median mean intensity-weighted lifetime values (same three replicates) obtained within 500–600 or 500–700 nm. For spectral imaging analysis (∼9 nm channels), we previously concluded selection of λ_Lo_ and λ_Ld_, 557 and 664 nm for NR12S and 583 and 673 nm for NR12A, respectively.^25^ At these wavelengths, the differences in the intensity of ordered and disordered phases generated the largest GP range while maintaining a sufficient fluorescence signal. Correspondingly, for the spectrally resolved FLIM data, we selected similar detection wavelengths (λ_Lo_ and λ_Ld_) for GP calculations, i.e., 550 and 670 nm for NR12S and 590 and 670 nm for NR12A, respectively. For Flipper, the values of 570 and 650 nm were chosen.

### Phasor Analysis

**Figure 2:**
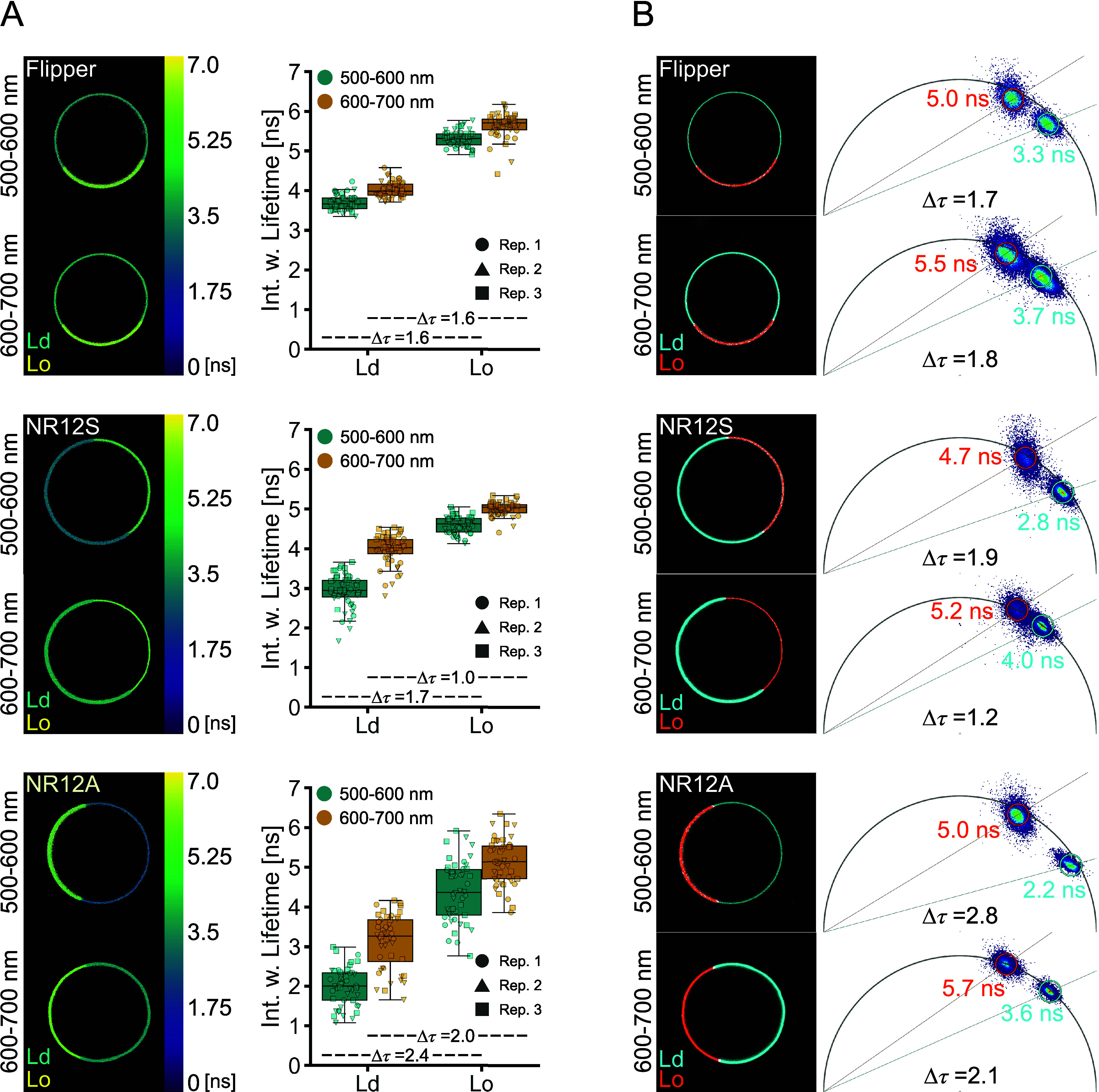
Lifetimes of Flipper, NR12S, and NR12A are sensitive to phase separation. Lifetime measurements of the probes in phase-separated GUVs were carried out at 500–600 or 600–700 nm emission. Multiexponential curve fitting was performed for the fluorescence decays. (A) Representative images of lifetime color-coded phase-separated GUVs and comparison of the intensity-weighted lifetime in liquid-disordered (Ld) vs liquid-ordered (Lo) phases at different emission wavelengths of Flipper (above), NR12S (middle), and NR12A (below). Different symbols correspond to GUVs of individual biological replicates (*n* = 3). Δτ values were calculated from the mean of the intensity-weighted lifetimes. (B) Phasor analysis of Flipper (above), NR12S (middle), and NR12A (below). Example images of lifetime separation (left) according to the clouds on the phasor plot (right) with Ld shown in cyan and Lo shown in orange. Δτ values were calculated from the average lifetimes of the phasor clouds.

Phasor analysis of lifetime was performed using LAS X FLIM/FCS software (version 4.5.0). To display the detected and time-sorted photons in the phasor plot, a wavelet filter with a threshold of 5 was applied, for better differentiation of the phasor photon clouds. For analysis, the center of the phasor photon clouds was manually selected with the circular selection tool of radius 20 (see Figure 2B). The average estimated fluorescence lifetime within the circle was used for further comparisons.

## Results and Discussion

Spectral imaging has shown that the environment-sensitive fluorescent probes NR12S and NR12A have different sensitivities to membrane biophysical properties such as the saturation index, cholesterol content, double bond position, and configuration as well as lipid headgroup charge and geometry.^25^ The shift in the probes’ emission spectrum in different lipid environments reflects these sensitivities (Figure 1A, see Figure S1 for structures and excitation spectra). Although Flipper is a lifetime-sensitive probe, it also exhibits minor shifts toward longer wavelengths of its emission spectrum in more disordered lipid environments (Figure 1A), which is in line with previous studies.^27^ As NR12S and NR12A are suitable for the investigation of lipid environments by spectral imaging, we aimed to examine their suitability for fluorescence lifetime analysis. Therefore, we characterized their lifetime behavior alongside the Flipper probe with FLIM.

### Lifetime Increases at Longer Emission Wavelengths

Regarding the fluorescence decays of all the probes in a disordered vs ordered (Δ9*cis* DOPC vs DPPC:Chol 50:50 LUVs) lipid environment, we observed a shift toward longer decay times in the ordered lipid environment (Figure 1B). Interestingly, we also observed a shift of the fluorescence decay toward longer times for all three probes in LUVs of the same lipid composition at longer wavelengths (Figure 1C). This shift is more pronounced in NR12S and NR12A compared to Flipper. Due to this observation, we further investigated the fluorescence decay time in 20 nanometer wavelength intervals from 500 to 700 nm (laser repetition rate of 20 MHz), to quantitatively evaluate the changes in lifetime at different emission wavelengths. We obtained the intensity-weighted fluorescence lifetime of all probes (from hereon referred to as lifetime) by exponential reconvolution fitting of the decay times (for details on data acquisition and curve fitting analysis, see the Methods and Table S1). The lifetimes of all three membrane probes in Δ9*cis* DOPC increase with longer emission wavelength, while a common control dye (AlexaFluor 488 in water) does not exhibit any such difference as a function of the collection window (Figure 1D). The increase in lifetime is less pronounced for Flipper, reaching a plateau already at around 600 nm compared to the lifetimes of NR12S and NR12A, which plateau at 650 nm. The observed emission wavelength dependency of the lifetimes of all probes is most likely caused by the solvent relaxation process. Upon excitation, the dipole moment of the fluorophore is rapidly reoriented, resulting in an energetically unfavorable Franck–Condon state.^38^ The molecules of the solvation envelope of the fluorophore then reorient, allowing the system to relax, thereby lowering the energy of the excited state. This reorientation of the solvent envelope takes time, which is reflected in a progressively increasing red-shift emission. The longer the fluorophore remains in the excited state, the longer the wavelength of the photons it emits.^38^ By splitting the overall fluorescence decay of the probes in 20 nm intervals in LUVs, we collected photons of short wavelength with short lifetimes from not fully relaxed fluorophores, which contrast photons of long wavelength with long lifetimes from fully relaxed fluorophores. The emission of Flipper is less affected by solvent relaxation^27^ compared to NR12S and NR12A as confirmed by detecting only minor shifts in its emission spectra in different lipid environments (Figure 1A). The detected emission wavelength dependency of the lifetimes was previously reported for Nile Red in different solvents.^32^ Therefore, an increase in lifetime in more red-shifted wavelengths is a potential indicator of the probes’ sensitivity for the solvent relaxation process.

### Flipper Lifetime Is Sensitive to the Cholesterol Content but Not Acyl Chain Order

We then examined the lifetimes of Flipper, NR12S, and NR12A in different lipid environments of varying cholesterol contents, saturation indices, double bond positions, and configurations as well as headgroups to investigate and quantify the probes’ lifetime variability and sensitivity.

The emission wavelength dependency of the lifetime of Flipper was observed in all lipid environments (Figure 1E and Figure S2). Flipper was shown to be able to differentiate between monounsaturated and saturated lipids (POPC:Chol 50:50 and DPPC:Chol 50:50) by an increase in lifetime Δτ = 0.47 ns (at 570 nm) (Figure 1E). However, among poly- or monounsaturated lipids (DAPC, Δ9*cis*DOPC, and POPC), the lifetime changes for Flipper are smaller Δτ = 0.31 ns (DAPC and Δ9*cis*DOPC at 570 nm). The double bond position and configuration (Δ6*cis*DOPC, Δ9*cis*DOPC and Δ9*trans*DOPC) as well as the headgroup charge and geometry (POPS and POPE) could hardly be differentiated by lifetime analysis (Figure S2). However, increasing amounts of cholesterol in the membrane result in increasing lifetime, which is in line with a previous study.^28^ Lipid packing and membrane tension are closely connected, as changed lipid composition (e.g., cholesterol introduction) as well as applied tension (e.g., micropipette aspiration) strongly influence lipid packing.^5,28^ This renders it almost impossible to distinguish the effect of membrane tension or lipid composition on the change of the lifetime of Flipper in model membrane systems,^28^ showing that fluorescent probes can be sensitive to multiple biophysical parameters.

### Lifetimes of NR12S and NR12A Are Highly Dependent on Emission Wavelength

Next, the lifetime sensitivity of NR12S and NR12A in different lipid environments was examined. The lifetime of NR12S increases in the presence of high cholesterol content with Δτ = 2.26 ns (POPC and POPC:Chol 50:50 at 570 nm) and upon switching from monounsaturated to saturated lipids with Δτ = 0.49 ns (POPC:Chol 50:50 and DPPC:Chol 50:50 at 570 nm) (Figures 1E and Figure S2). The sensitivity to cholesterol is in line with previous results both in model membranes (LUVs)^39^ and more complex membranes (neuronal membranes of the hippocampus).^40^ Interestingly, these two lipid compositions are the only ones in which the emission wavelength dependency of the lifetime of NR12S was much less pronounced. While the lifetime differences of NR12S at shorter emission wavelengths (550 and 610 nm) are quite prominent, they are much smaller at longer emission wavelengths (630–690 nm). Similar to NR12S, NR12A can only distinguish lipid compositions of high cholesterol content from the other lipid compositions with Δτ = 2.06 ns (POPC and POPC:Chol 50:50 at 570 nm) and saturated from monounsaturated lipids with Δτ = 1.20 ns (POPC:Chol 50:50 and DPPC:Chol 50:50 at 570 nm) (Figures 1E and S2). Among the three probes, the lifetime of NR12A shows the most pronounced emission wavelength dependency: while the lifetime of NR12A can distinguish the two high-cholesterol lipid compositions across the entire emission spectrum, the sensitivity is better (larger lifetime changes) at shorter emission wavelength regions (550–610 nm). As mentioned above, the strong emission wavelength dependency of NR12S and NR12A is due to solvent relaxation. Additionally, Nile Red-derived dyes exhibit complex photophysical behavior including a twisted internal charge transfer (TICT),^32,41,42^ which competes with solvent relaxation, causing multiple emitting species affected by the TICT with varying fluorescence properties, as observed in time-resolved emission spectrum (TRES) experiments of NR12S and NR12A.^25^ Of note, fitting of the decay curves of NR12S and NR12A at longer wavelength regions (630–690 nm) was less accurate compared to Flipper, indicated by the χ^2^ values (Figure S3).

### Optimal Laser Repetition Rate Is Crucial for FLIM Measurements

To investigate the effect of parameter fitting, we also studied the hardware performance in our FLIM system. Notably, the frequency of the pulsed laser has a big impact on the reliable lifetime values. We observed that laser excitation frequencies of 40 or 80 MHz cause an overestimation of fluorescence lifetimes and less reliable fitting (indicated by χ^2^ values), especially in the longer emission wavelength region and more ordered lipid compositions (NR12A as an example, Figure S4A). Looking more closely at the fluorescence decay curves and the fitting performance, the offset increases with rising frequency as the decay is not completed within the interval between two laser pulses (t-repeat), and the accuracy of the fit decreases, indicated by the increasing residuals around the laser pulse (NR12S in DPPC:Chol 50:50 as an example, Figure S4B). In lifetime analysis (via fitting time-correlated single photon counting histograms), a pile-up effect occurs at high photon count rates, where photons with short arrival times are over-represented due to the detector dead times, resulting in an overall underestimation of lifetimes.^30^ We therefore strongly recommend selecting a laser repetition rate that is low enough to allow for complete fluorescence decays and for intervals between pulses (t-repeat) to be around 10 times longer than the average lifetime. For example, for an average lifetime of 5 ns, we suggest a time between two pulses of 50 ns equal to a laser repetition rate of 20 MHz or shorter. We further suggest adjusting the laser intensity to achieve fluorescence emission detection rates of less than one photon per pulse. Our conclusions are also in line with another study examining the laser frequency influence on the lifetime of Flipper in more detail.^43^

### Lifetimes of NR12A and NR12S Distinguish Membrane Phases Better at the Green Spectral Region

Lateral heterogeneity within membranes is crucial for their varying functions.^6^ While disordered phases are characterized by mostly unsaturated lipids, ordered phases are enriched in saturated lipids and cholesterol.^5^ As revealed above, all probes show lifetime sensitivity to high cholesterol content, and we thus wanted to examine whether the emission wavelength dependency of the lifetime persists in other membrane systems and whether liquid-ordered (Lo) and liquid-disordered (Ld) phases are separated equally well at different spectral regions.

Therefore, we measured lifetimes of the probes in phase-separated GUVs (sphingomyelin:DOPC:Chol 2:2:1). Although spectrally fine-resolved lifetime measurements give a more detailed insight into plasma membrane properties, the approach was not feasible for the use in larger model membrane systems or cells due to collection constraints of detected photons (i.e., photon budget). For reliable multicomponent exponential fit analyses, a high number of detected photons are crucial (10^2^, 10^4^, and 10^6^ for mono-, bi-, and triexponential fitting, respectively)^30,37^ in each emission window, which requires high laser power, multiple frame repetitions, and almost completely immobile samples. Instead of narrow spectral bands, we therefore collected photons in two wider channels of equal wavelength, separating the extreme peaks in the emission spectrum (Figure 1A): at shorter (550 ± 50 nm; referred to as green) and longer (650 ± 50 nm; referred to as red) wavelengths (Figure 2A). Moreover, to minimize the contribution of noise and obtain the needed photon counts for fitting analysis, we selected regions of interest (ROIs) of the Lo and Ld phases separately, instead of performing pixel-wise fitting (Figure S5A). The same selection was used for lifetime analysis in the green and red channels. All three probes can differentiate Lo from Ld phases by an increase in lifetime in both emission windows, with higher lifetimes in red (Figure 2A), confirming the experiments in LUVs discussed above. Flipper differentiates the phases equally well in both emission windows with a shift in the lifetime of Δτ = 1.6 ns, which is in line with previous investigations^28^ and *in silico* studies.^44^ The Lo labeling of Flipper seems to be more prominent, which has been previously explained by the increased oscillator strength in its more planarized form, which yields more photons.^27^ In contrast, NR12S and NR12A exhibit a higher lifetime resolution for the membrane phases in the green with Δτ = 1.7 and 2.4 ns compared to Δτ = 1.0 and 2.0 ns in the red, respectively. This is consistent with our experiments performed in LUVs. At shorter wavelengths, NR12S and NR12A showed higher Δτ between Lo and Ld; however, the heterogeneity of their lifetime is higher than that of Flipper. Fitting of the decay curves according to the χ^2^ values was quite reliable for all three probes with some outliers occurring for NR12S and NR12A (Figure S5B).

### Phasor Analysis of Lifetime Distinguishes Membrane Phases Better at the Green Spectral Region

As a fit-free technique, phasor analysis of Fourier-transformed fluorescence decays generates lifetime maps of pixel-detected photon arrival times in a 2D phasor plot.^30,45^ Fluorescent probes with a monoexponential decay locate on the universal semicircle in these plots, whereas probes with multiexponential decaying properties locate within the universal semicircle.^45^

To complement our decay fitting approach, we employed phasor analysis to investigate the lifetime of the probes in the Lo and Ld phases (Figure 2B). First, the location of the phasor clouds indicates the lifetime complexity: Flipper locates within the semicircle irrespective of the membrane phase or detection window, indicating a multiexponential decay in line with the performed biexponential curve fitting and previous studies.^28^ NR12S locates within the semicircle at shorter wavelengths but locates on the edge of the semicircle at longer wavelengths, indicating a multiexponential decay at shorter and a monoexponential decay at longer wavelengths. For NR12A, only the Lo phase at longer wavelengths is located on the semicircle, indicating a monoexponential decay. All other phasor clouds are located within the semicircle, thus indicating multiexponential decays. The observation of the distribution of mono- and multiexponential decays of NR12S and NR12A agrees with the exponential curve fitting analysis (Table S2). Previous studies with NR12S used a biexponential fitting analysis, in line with our results.^39,40^ Concerning fit-free phasor analysis, all probes can easily separate the phases, as seen by two distinct clouds in the phasor plots. The shift in lifetimes obtained from the phasor analysis follows a similar trend in both spectral windows as seen above, further confirming the emission wavelength dependency. NR12S and NR12A again show better separation at the green spectral region with Δτ = 1.9 ns and Δτ = 2.8 ns compared to the red spectral region with Δτ = 1.2 ns and Δτ = 2.1 ns, respectively (inset phasor plots Figure 2B).

### Lifetime Imaging in Cells Confirms Wavelength Dependency

**Figure 3:**
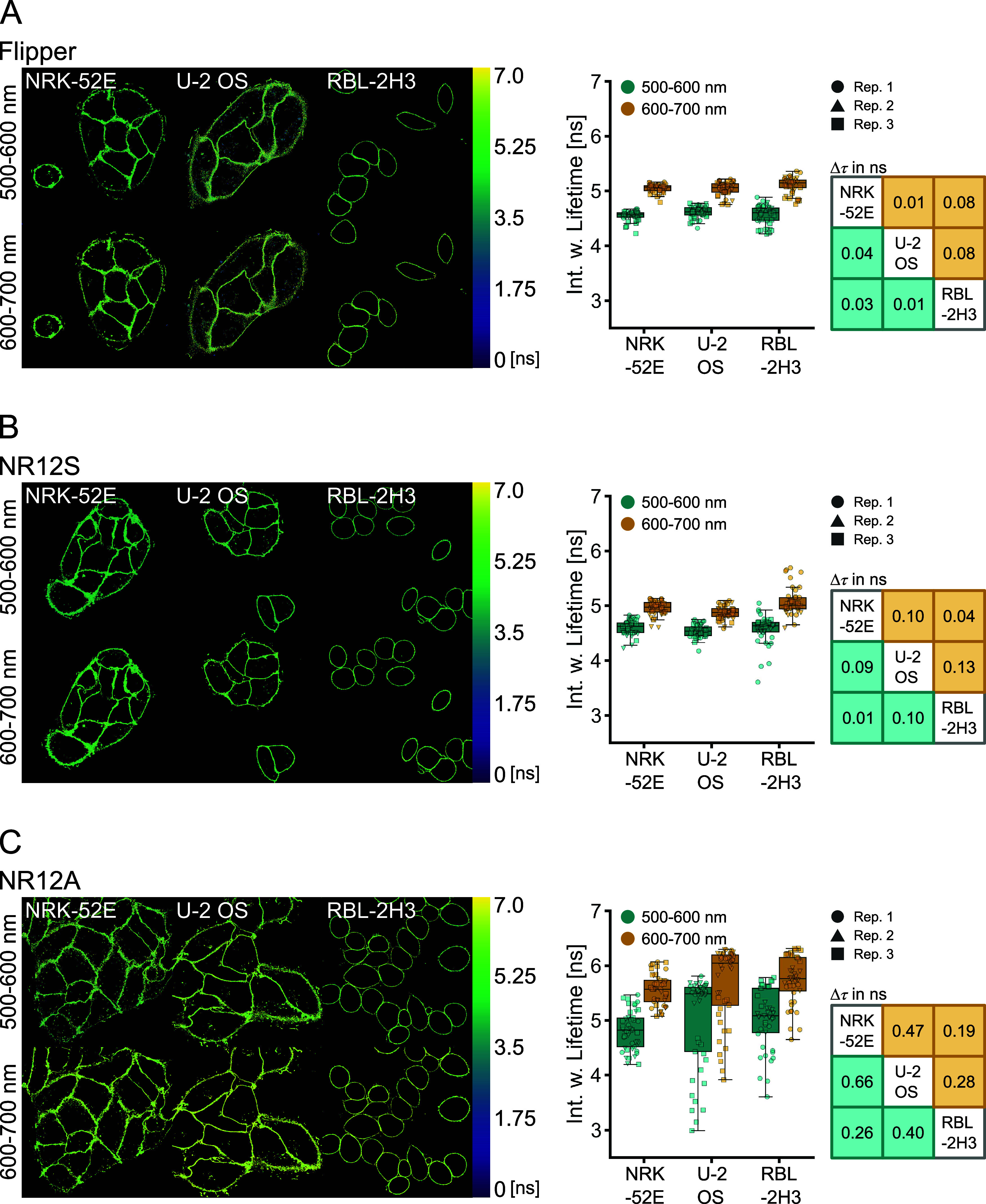
Lifetimes of Flipper, NR12S, and NR12A in different cell types. Lifetime measurements of the probes in NRK-52E, U-2 OS, and RBL-2H3 cells were carried out at 500–600 and 600–700 nm emission windows. Multiexponential curve fitting was performed for the whole-image fluorescence decays. (A) Representative images of lifetime color-coded cells stained with Flipper and comparison of the intensity-weighted lifetime in different cell types and at different emission wavelengths. (B) Representative images of lifetime color-coded cells stained with NR12S and comparison of the intensity-weighted lifetime in different cell types and at different emission wavelengths. (C) Representative images of lifetime color-coded cells stained with NR12A and comparison of the intensity-weighted lifetime in different cell types and at different emission wavelengths. Different symbols correspond to images of individual biological replicates (*n* = 3). Tables indicate Δτ values of the medians between the cell types in the two channels.

Next, to evaluate the applicability of these probes with lifetime analysis in complex cellular environments, we used different adherent cell lines, NRK-52E, U-2 OS, and RBL-2H3 (Figure 3). Again, due to photon budget constraints, photons were collected in the broader green and red channels, similar to the phase-separated GUVs. Given that the membrane probes are fluorogenic (i.e., only fluoresce upon integration into the membrane^11^), the cells were imaged directly after staining to avoid internalization during image acquisition. Longer incubation times with Flipper have shown internalization into endocytic compartments, displaying a shorter lifetime, thus affecting the overall lifetime in whole-image analysis,^43^ which is why we kept incubation times short (see the Methods). The emission wavelength dependency with longer lifetimes at longer wavelengths was again observed for all three probes, in line with the results in model membranes. Flipper and NR12S do not show large lifetime shifts among the different cell types (Figure 3A,B).

Similar to lifetime measurements in LUVs and GUVs, Flipper lifetime shifts are within the same range at shorter and longer emission wavelengths (Δτ < 0.1 ns). This is also the case for NR12S (Δτ < 0.15 ns), contrasting experiments in simpler lipid environments, where the lifetime resolution was better at shorter wavelengths. Lifetime analysis of NR12A resulted in very heterogeneous lifetime values for U-2 OS and RBL-2H3 cells, which obscure resolving differences in membrane biophysical properties (Figure 3C). Fitting analysis for NR12A was less reliable compared to Flipper and NR12S, indicated by increasing χ^2^ values, especially at longer detected emission wavelengths (Figure S6), which could explain the lifetime heterogeneity. To obtain sufficient photon counts for multiexponential curve fitting of the decay and avoid phototoxicity due to long acquisition times in live cells, we performed whole-image analysis instead of pixel-wise fitting. This approach may, on the other hand, average out small differences, emphasizing a limitation of FLIM analysis with these probes in complex environments.

### Lifetimes of Flipper and NR12A Distinguish Delta-Spike VLPs

**Figure 4:**
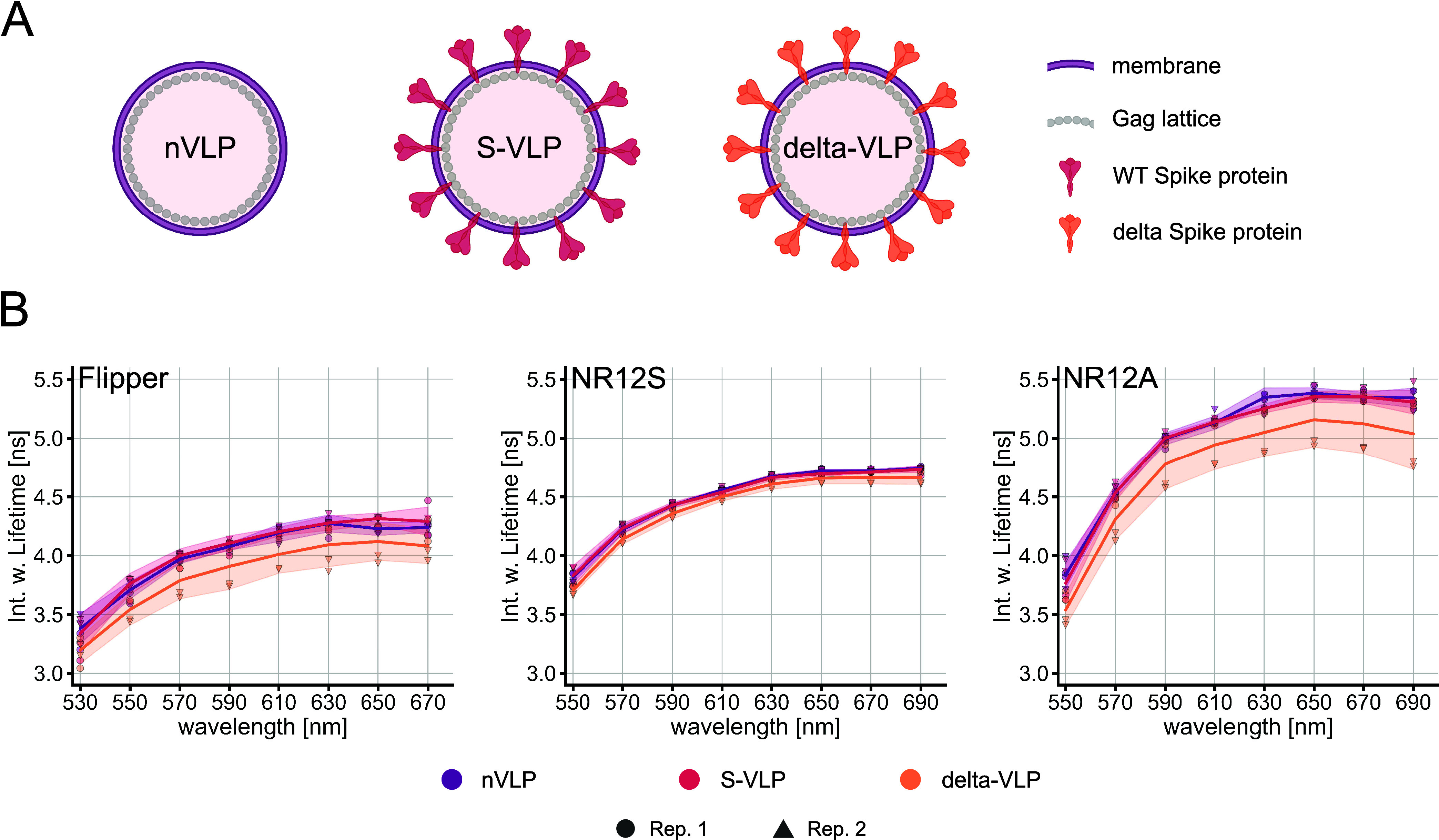
Lifetimes of Flipper, NR12S, and NR12A in different VLP species. (A) Schematic representation of the SARS-CoV-2 n-VLPs, S-VLPs, or delta-VLPs (created with Biorender.com). (B) Spectral fluorescence lifetime measurements of the probes in the VLPs were carried out within 500–700 nm in intervals of 20 nm. Multiexponential curve fitting was performed for the fluorescence decays. Spectrally resolved intensity-weighted lifetime of Flipper (left), NR12S (middle), and NR12A (right) in different VLP species. The line corresponds to the median of individual biological replicates shown with different symbols (*n* = 2). The band corresponds to standard deviation.

The virus envelope is a complex structure where environment-sensitive probes are helpful to understand the biophysics of host–pathogen interactions. Virus-like particles have wide application in biology and medicine, ranging from unraveling viral structures and investigation of virus–host cell interactions to vaccine development.^46^ Viruses with a lipid envelope obtain the lipids from the host cell plasma membrane upon virus budding. During the budding process, lipid–protein interactions drive the accumulation of certain lipids into the viral envelope,^47^ resulting in varying envelope lipid compositions of different viruses with potentially distinctive biophysical properties. Especially, SARS-CoV-2 spike variants evolved to have increased positive charge, resulting in enhanced binding.^48^ Whether these severe surface changes are accompanied by alterations in the biophysical properties of the viral membrane is unknown to date. Therefore, we investigated the lifetimes of the probes in naked HIV-1 Gag-based VLPs or pseudotyped with SARS-CoV-2 WT and delta-spike protein (nVLP, S-VLP, delta-VLP) (Figure 4A). Similar to the LUV measurements, the lifetimes were examined in 20 nm windows.

Our results indicate that Flipper and NR12A can differentiate membrane properties of delta-VLPs compared to other investigated VLPs by a lifetime shift to lower values with Δτ = 0.18 and 0.19 ns (deltaVLPs and nVLPs at 610 nm), respectively. However, both probes exhibit a higher standard deviation of the lifetimes in delta-VLPs. The lifetime of NR12S is hardly sensitive to the different VLP types with Δτ = 0.06 ns (deltaVLPs and nVLPs at 610 nm) (Figure 4B). The emission wavelength dependency is again observed for the VLP measurements of all three probes, while it is less pronounced compared to the liposomes. Fitting analysis was quite reliable for all probes indicated by χ^2^ values (Figure S7).

Viral membranes comprise different lipid species and are known to be highly enriched in sphingomyelin and cholesterol (up to 50%).^49−52^ In fact, the lifetimes of the probes in the different VLP species resemble the lifetimes in POPC with 50% cholesterol. Lifetimes of Flipper and NR12A are more sensitive to a high cholesterol content than that of NR12S, indicating that there might be a slightly lower cholesterol content in delta-VLPs, which is not detectable by NR12S. A previous study using NR12S did not observe detectable differences in the diffusion or GP value between nVLPs and delta-VLPs.^35^ In addition to cholesterol sensitivity, Flipper also reports on membrane tension, indicating that delta-VLPs might also exhibit distinct membrane tension. Application of membrane tension in single lipid species membranes results in a lower lifetime due to lipid decompression, which gives Flipper more space to relax into its more twisted conformation.^27^ However, viral membranes are heterogeneous, in which application of external membrane tension (not caused by increased lipid packing due to cholesterol incorporation) results in higher lifetimes due to membrane reorganization.^27^ Taken together, slight alterations in the cholesterol content and/or an increase in membrane tension potentially explains the observed decrease in the lifetimes of NR12A and Flipper in delta-VLPs, respectively. Nevertheless, future studies should focus on determination of multiple parameters (e.g., fluidity, tension, viscosity, and charge among others) at once, to better describe the biophysical profiles of viral membranes.

### Which Analysis Method Should Be Used for NR12S and NR12A: Ratiometric GP or Fluorescence Lifetime?

**Figure 5:**
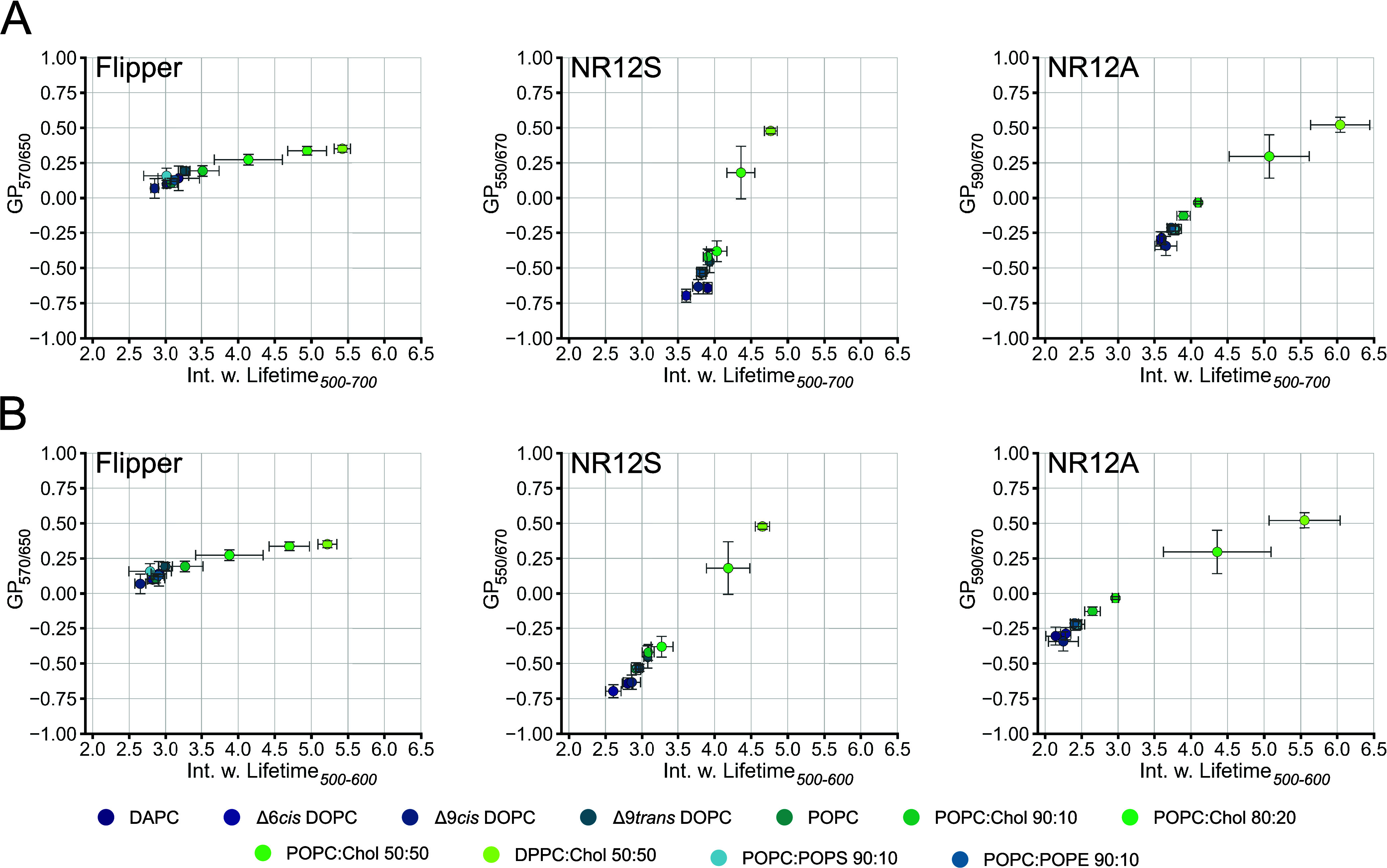
GP vs lifetime from Flipper, NR12S, and NR12A in different lipid environments. Spectral fluorescence lifetime measurements of the probes in LUVs were carried out within 500–700 nm in intervals of 20 nm. Multiexponential curve fitting was performed for the fluorescence decays within (A) 500–700 nm or (B) 500–600 nm wavelength ranges. Comparison of resolution power of Flipper (left), NR12S (middle), and NR12A (right) in intensity-based GP analysis vs lifetime analysis in different lipid environments. Dots correspond to the median GP of the respective wavelengths and median mean intensity-weighted lifetime. Error bars correspond to the standard deviation. GP and lifetime analysis was performed using the same dataset.

Given that NR12S and NR12A are mainly used for intensity-based ratiometric analysis (GP), we wanted to compare the resolution powers of lifetime vs GP for all three probes in LUVs of different lipid compositions (Figure 5). The wavelengths for the GP calculation were chosen to give high GP resolution while maintaining sufficient signal intensity (see the Methods). As expected, Flipper shows high sensitivity for different lipid compositions by lifetime analysis with Δτ = 2.57 ns (Δ9*cis*DOPC and DPPC:Chol 50:50 from 500–700 nm), compared to GP analysis with ΔGP = 0.28 (Δ9*cis*DOPC and DPPC:Chol) (Figure 5A), due to its minor shifts of its emission spectrum (Figure 1A). When the whole spectrum is used for lifetime estimation (mean intensity-weighted lifetime within 500–700 nm) for NR12S and NR12A, ratiometric GP analysis (ΔGP = 1.11 for NR12S and ΔGP = 0.86 for NR12A in Δ9*cis*DOPC and DPPC:Chol 50:50) is clearly advantageous in differentiating different lipid compositions over lifetime-based analyses (Δτ = 0.99 ns for NR12S and Δτ = 2.39 ns for NR12A) (Figure 5A). However, adjusting the detection window to shorter emission wavelengths (green channel, 500–600 nm), the lifetime resolution especially in more unsaturated, low-cholesterol lipid environments increases dramatically (Δτ = 1.79 ns for NR12S and Δτ = 3.30 ns for NR12A in Δ9*cis*DOPC and DPPC:Chol 50:50) (Figure 5B). In contrast, the lifetime resolution for Flipper remains unchanged at Δτ = 2.57 ns (Δ9cisDOPC and DPPC:Chol 50:50). These results highlight that the suitability of the lifetime-based measurements of NR12S and NR12A for membrane fluidity measurements strongly depends on the emission detection settings. Ultimately, using both lifetime and intensity information together, the separation of different membrane environments could further improve.

## Conclusions

Our understanding of the biophysical properties of cellular membranes has greatly benefited from environment-sensitive fluorescent probes. An increasing number of probes have been developed and are being constantly improved in their functional abilities regarding membrane localization and leaflet selectivity^12,16,17,19^ as well as photostability and brightness, allowing even for super-resolution imaging.^18^ Furthermore, their individual sensitivities to varying biophysical properties are being explored.^24,25,28^ Some of these probes have been modified for organelle membrane specificity, allowing the dissection of biophysical properties of different cellular compartments.^29,53^ While many of the solvatochromic probes have been investigated and characterized by spectral intensity imaging and subsequent GP analysis,^21,25^ the aim of this work was to investigate the probes NR12S and NR12A alongside Flipper in different lipid environments utilizing FLIM measurements. We explored the sensitivity of the lifetime in low-complexity model membrane systems as well as high-complexity live cells and VLPs.

Our work reveals that the lifetimes of NR12S and NR12A are mostly sensitive to larger changes in the cholesterol content and saturated versus monounsaturated lipid content, reliably differentiating Lo from Ld phases. Further, the lifetime of NR12A reports on different VLP species. Both probes show a large increase in lifetime with emission wavelength, likely due to solvent relaxation effects and complex photophysical behavior. The mechanism underlying lifetime shifts in different lipid compositions is most likely also solvent relaxation, as disordered membranes are interspersed with more water molecules, facilitating solvent relaxation, thus resulting in a shorter lifetime for disordered membranes and vice versa for ordered membranes.^38^ The location and orientation of the probes within the membrane also have a big impact on solvent relaxation as the immediate environment of the fluorescent moiety is different, resulting in distinct solvent relaxation processes and consequently variations of fluorescence lifetimes. In our previous study, by using atomistic molecular dynamics simulations, we showed that NR12S and NR12A assume different orientations and locations along the bilayer normal, which causes the probes to have different relaxation processes examined by time-dependent fluorescence shift analyses.^25^ This is also in line with another study, which found NR12A to be located close to the membrane–water interface at ∼18 Å from the bilayer center.^39^ On the other hand, the mechanism of Flipper’s lifetime variations is mainly based on conformational changes but also susceptible to solvent relaxation. Consequently, Flipper has a less pronounced emission wavelength dependency as it is also located deeper in the membrane among the lipid tails^44^ compared to NR12A and NR12S. Using FLIM, we observed that Flipper is primarily sensitive to the cholesterol content rather than the acyl chain order, can reliably distinguish different membrane phases, and reports on different VLP species. The underlying mechanistic principle of variations in the lifetime of Flipper is its planarization upon membrane tension or changes in lipid composition (e.g., cholesterol incorporation) resulting in altered lipid packing.^27,28^ We cannot exclude an influence of the acyl chain order on Flipper lifetime as we did not investigate highly ordered lipid compositions without cholesterol. In fact, molecular dynamics simulations show that Flipper strongly planarizes in the DPPC gel phase,^44^ suggesting an expected lifetime increase under these conditions. Of note, in complex heterogeneous membranes, the impact of either lipid composition or membrane tension is not at all trivial to dissect.

Moreover, we emphasize that although FLIM allows an intensity-independent readout, fluorescence lifetime measurements, subsequent analysis, and interpretation with these environment-sensitive membrane probes are not trivial. For reliable fitting of the fluorescence decay, a sufficient photon count rate and the adequate laser frequency must be provided to avoid technical pitfalls in experiments. For fluorescent probes with multiexponential decays, which is true for all three probes used in this study, high photon counts need to be collected for optimal analysis. The technical concerns imply a need for high laser intensities and result in increasing phototoxicity in live samples and bleaching of the probes. Lower intensities generate technical concerns for long acquisition times, which are problematic for moving specimens and probe internalization. To unravel potential small-scale heterogeneities in more complex biological systems, pixel-wise lifetime fitting is required. However, a pixel-wise fit is challenging due to the photon budget constraints of these probes, which results in lifetime averaging analysis (image area selection). Fit-independent analysis applying phasor plots can be explored here to group average lifetime components in an image. Furthermore, not all FLIM setups are equipped with pulse pickers allowing adjustment of the laser frequency. Full exploration of lifetime dynamics across different emission spectra could result in an additional need for high-cost equipment if reliable curve fitting is to be expected. Moreover, the spectrally dependent detector sensitivity should be considered when collecting photons across a wide wavelength interval, as it can lead to an underestimation of the intensity-weighted lifetime calculated from multiexponential decays. This can be avoided by measuring in short wavelength intervals or at wavelength intervals at which the quantum efficiency of the detector is relatively stable.

The quantified emission wavelength dependency exhibited by all three membrane probes in this study indicates possibilities and concerns, especially regarding spectral settings in lifetime measurements. This effect is especially true for NR12S and NR12A as their lifetime resolution largely increases at shorter wavelengths (500–600 nm) compared to longer wavelengths or the full spectrum. We emphasize that for comparative lifetime studies, the same detection wavelength or wavelength interval across the different membrane samples should be used. Therefore, instead of using the detection wavelengths at the fluorescence intensity maximum, we suggest wavelength intervals that provide the best lifetime resolution between the different samples while still maintaining sufficient intensity (photon count) for reliable lifetime analysis. Moreover, our detected wavelength dependency should be investigated for other membrane probes commonly used for lifetime analysis, such as Di-4-ANEPPDHQ or Laurdan. Increased lifetime resolution of these dyes might be achieved by optimizing the detection wavelength. Seeing more complex multiexponential decays of investigated probes at shorter wavelengths, compared to monoexponential decays at longer wavelengths, could also be used beneficially or with concern.

In this study, we primarily investigated the lifetime sensitivity of the probes NR12S, NR12A, and Flipper to membrane fluidity. The biophysical properties of the membrane such as fluidity, viscosity, and tension strongly influence each other.^54^ Therefore, further studies should investigate the sensitivity of the probes to other biophysical properties, such as viscosity. Further, our experimental pipeline can also be applied to study viscosity-sensitive probes, such as molecular rotors.

In summary, we provided an in-depth analysis of the lifetime behavior of the probes Flipper, NR12S, and NR12A in different lipid environments of model membranes as well as physiological membranes. Further, we emphasize the important factors regarding data acquisition and analysis to be kept in mind when applying these probes in different biological contexts using FLIM.

## Data Availability

All data are available upon publication in FigShare DOI: 10.17044/scilifelab.25186151.

## Supporting Information Available

Acquisition and fitting parameters for all FLIM measurements, structures and excitation spectra of the probes, probe lifetimes in different lipid compositions, Chi-squared values of the curve fitting analysis, laser frequency influence, and phase selection in phase-separated GUVs (PDF)
