# Supplementary material for: Quantifying Fluorescence Lifetime Responsiveness of Environment-Sensitive Probes for Membrane Fluidity Measurements
Source: J Phys Chem B. 2024 Feb 28;128(9):2154–67. doi: 10.1021/acs.jpcb.3c07006 (PMC10926104; doi:10.1021/acs.jpcb.3c07006)
Supplement: Supplementary file 1 — jp3c07006_si_001.pdf [file jp3c07006_si_001.pdf]

Supporting Information

Quantifying Fluorescence Lifetime Responsiveness of Environment-Sensitive Probes for Membrane Fluidity Measurements

Franziska Ragaller[1], Ellen Sjule[1], Yagmur Balim Urem[1], Jan Schlegel[1], Rojbin El[2], Dunja Urbancic [2,3], Iztok Urbancic[4], Hans Blom[5], Erdinc Sezgin[1,*]

[1]Science for Life Laboratory, Karolinska Institutet, Department of Women's and Children's Health, 17165, Solna, Sweden

[2]Weatherall Institute of Molecular Medicine, University of Oxford, OX39DS, Oxford, United Kingdom

[3]Faculty of Pharmacy, University of Ljubljana, SI-1000 Ljubljana, Slovenia

[4]Laboratory of Biophysics, Condensed Matter Physics Department, Jožef Stefan Institute, Jamova Cesta 39, 1000 Ljubljana, Slovenia

[5]Science for Life Laboratory, Department of Applied Physics, Royal Institute of Technology, 17165, Solna, Sweden.

Correspondence:

Erdinc Sezgin

erdinc.sezgin@ki.se

Phone: 0046702318248

Table S1: Acquisition parameters for all FLIM measurements.

	probe	laser intensity	frame repetitions	line repetitions	scan speed	detector	emission
LUVs	Flipper (1 µM)	20%	10 - 20	1	400 Hz	SMD HyD (10% Gain) photon-counting	500-700 nm in 20 nm intervals (sequentially)
	NR12S (1 µM)	15%	10	1			
	NR12A (1 µM)	15%	10	1			
	AF488 (1 µM)	2%	10	1			
phase-separated GUVs	Flipper (300 nM)	5% (500-600 nm) 10% (600-700 nm)	1	4	200 Hz		500-600 nm & 600-700 nm (sequentially)
	NR12S (100 nM)	5%	1	2			
	NR12A (100 nM)	5%	1	2			
cells	Flipper (1 µM)	3-5%	1	4	200 Hz		500-600 nm & 600-700 nm (sequentially)
	NR12S (1 µM)	1%	1	4			
	NR12A (1 µM)	0.2 -1 %	1	4			
VLPs	Flipper (300 nM)	15%	2	16	400 Hz		500-700 nm in 20 nm intervals (sequentially)
	NR12S (100 nM)	15%	2	16			
	NR12A (100 nM)	15%	2	16			

Table S2: Fitting parameters for all FLIM measurements.

	analysis area	laser frequency	fitting range	probe	number of fitting components	environment
LUVs	whole image analysis	20 MHz	0.2-45 ns for each 20 nm window	Flipper	2 components: 500-700 nm	all lipid compositions
				NR12S	2 components: 500-620 nm<br>1 component: 620-700 nm	DAPC, Δ6*cis* DOPC, Δ9*cis* DOPC, Δ9*trans* DOPC, POPC, POPC:Chol 90:10, POPC:Chol 80:20, POPS, POPE
					2 components: 500-600 nm<br>1 component: 600-700 nm	POPC:Chol 50:50, DPPC:Chol 50:50
				NR12A	2 components: 500-640 nm<br>1 component: 640-700 nm	DAPC, Δ6*cis* DOPC, Δ9*cis* DOPC, Δ9*trans* DOPC, POPC, POPC:Chol 90:10, POPS, POPE
					2 components: 500-620 nm<br>1 component: 620-700 nm	POPC:Chol 80:20, POPC:Chol 50:50, DPPC:Chol 50:50
				AF 488	1 component: 500-700 nm	in water
			0.2-45 ns from 500-600 nm or over whole spectrum	Flipper	2 components	all lipid compositions
				NR12S	2 components	all lipid compositions
				NR12A	2 components	all lipid compositions
		40 MHz	0.2-25 ns for each 20 nm window	NR12A	2 components: 500-640 nm<br>1 component: 640-700 nm	Δ9*cis* DOPC, POPC
					2 components: 500-620 nm<br>1 component: 620-700 nm	DPPC:Chol 50:50
		80 MHz	0.2-12.5 ns for each 20 nm window	NR12A	2 components: 500-640 nm<br>1 component: 640-700 nm	Δ9*cis* DOPC, POPC
					2 components: 500-620 nm<br>1 component: 620-700 nm	DPPC:Chol 50:50

	analysis area	laser frequency	fitting range	probe	number of fitting components	environment
**phase-separated GUVs**	region of interest selection	20 MHz	0.2-45 ns for each 100 nm window	Flipper	2 components: 500-700 nm	Ld and Lo phase
				NR12S	3 components: 500-600 nm<br>1 component: 600-700 nm	Ld phase
					2 components: 500-600 nm<br>1 component: 600-700 nm	Lo phase
				NR12A	3 components: 500-600 nm<br>2 components: 600-700 nm	Ld phase
					3 components: 500-600 nm<br>1 component: 600-700 nm	Lo phase
**cells**	whole image analysis	20 MHz	0.2-45 ns for each 100 nm window	Flipper	3 components: 500-700 nm	all cell types
				NR12S	3 components: 500-600 nm<br>2 components: 600-700 nm	all cell types
				NR12A	3 components: 500-600 nm<br>1 component: 600-700 nm	all cell types
**VLPs**	whole image analysis	20 MHz	0.2-45 ns for each 20 nm window	Flipper	2 components: 500-700 nm	all VLPs
				NR12S	2 components: 500-620 nm<br>1 component: 620-700 nm	all VLPs
				NR12A	2 components: 500-620 nm<br>1 component: 620-700 nm	all VLPs

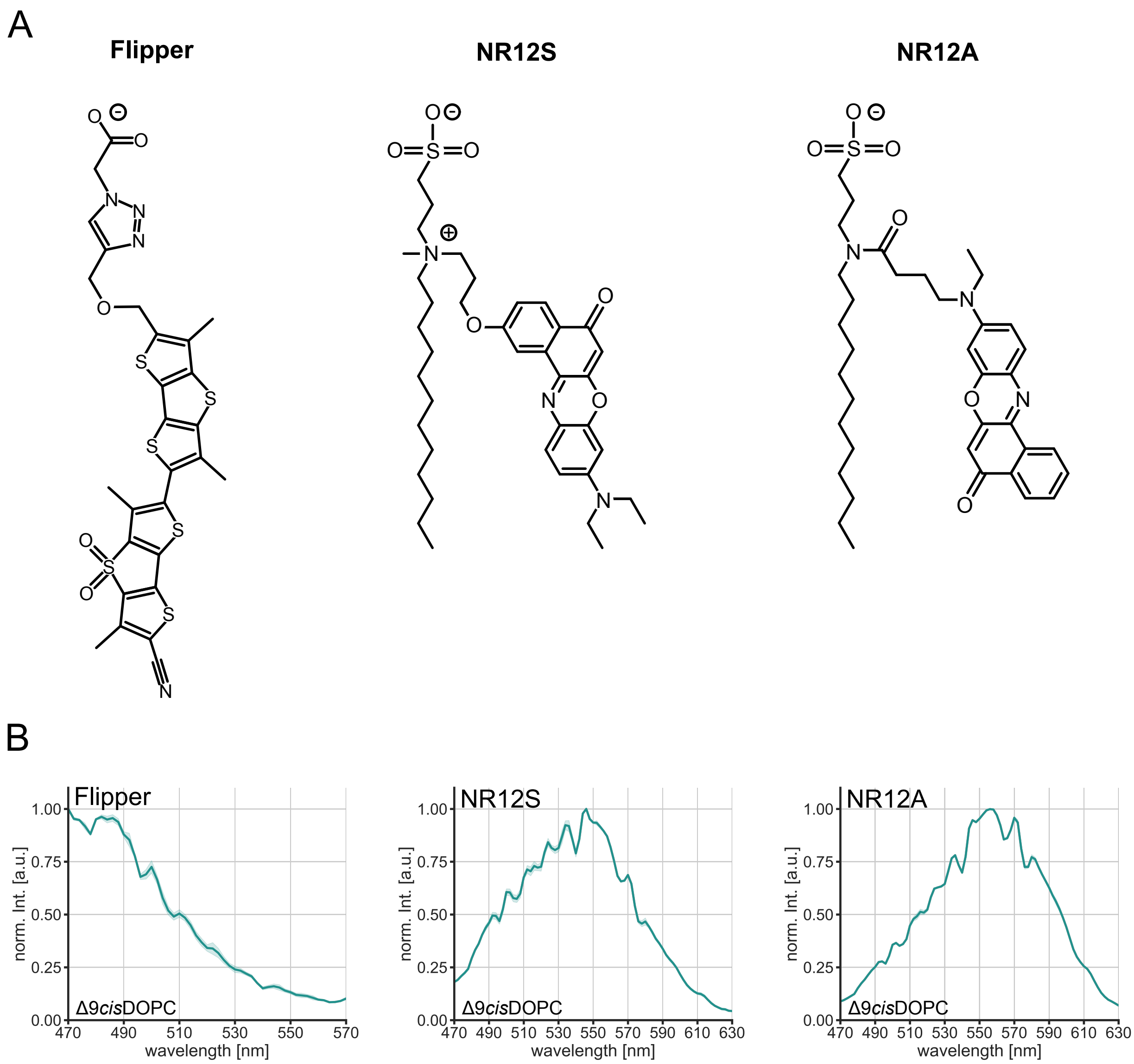

Figure S1: Chemical structures and excitation spectra of Flipper, NR12S and NR12A. A| Chemical structures of Flipper, NR12S and NR12A. B| Excitation spectra of the three probes (1 μM) in Δ9*cis*DOPC LUVs corrected for background signal. Line corresponds to the mean of three technical replicates. Band corresponds to the standard deviation.

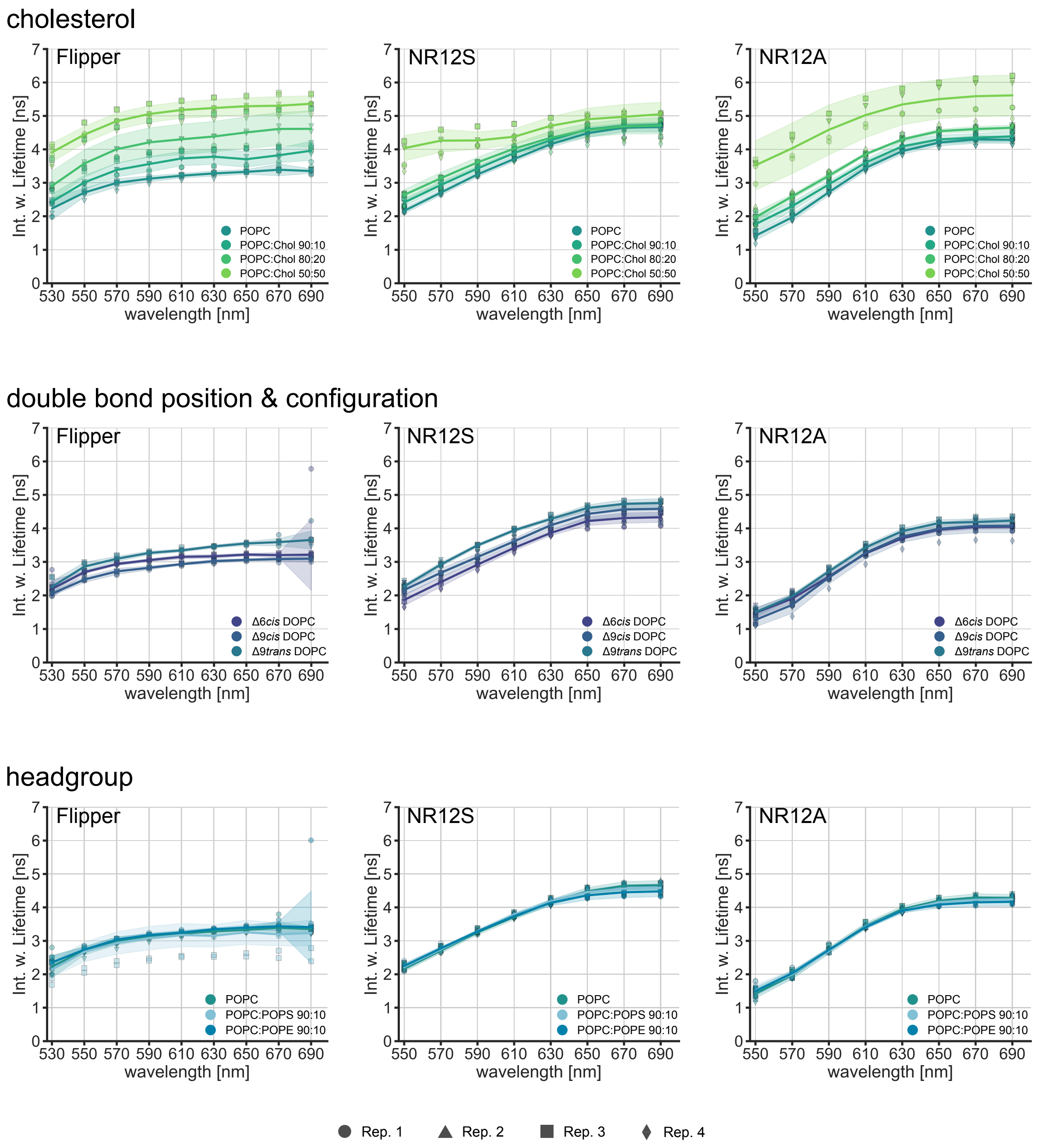

Figure S2: Lifetimes of Flipper, NR12S and NR12A in different lipid environments. Spectral fluorescence lifetime measurements of the probes in LUVs were carried out within 500-700 nm in intervals of 20 nm. Multiexponential curve fitting was performed for the fluorescence decays (for details see Material and Methods). Spectrally resolved intensity weighted lifetime of Flipper (left), NR12S (middle) and NR12A (right) in different lipid environments investigating cholesterol content (above), double bond position and configuration (middle) and headgroup geometry and charge (below). Line corresponds to the median of individual biological replicates shown with different symbols (n≥3). Band corresponds to standard deviation.

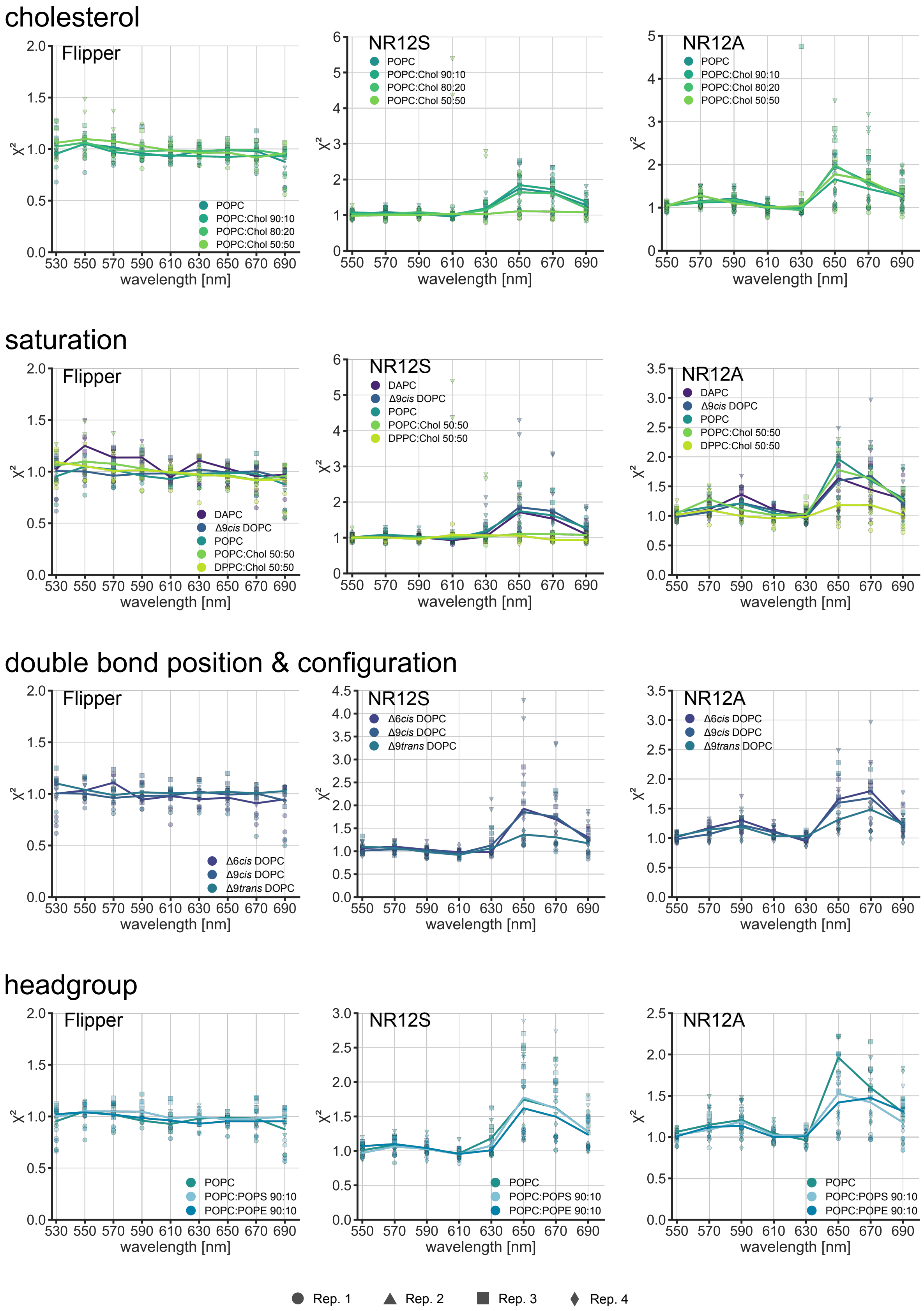

Figure S3: Chi-squared values of the multiexponential curve fitting of Flipper, NR12S and NR12A in different lipid environments.

Spectral fluorescence lifetime measurements of the probes in LUVs were carried out within 500-700 nm in intervals of 20 nm. Multiexponential curve fitting was performed for the fluorescence decays (for details see Material and Methods). χ^2 values serve as indicator for the goodness of the fit and were obtained for each 20 nm interval and are shown for Flipper (left), NR12S (middle) and NR12A (right) in different lipid environments investigating cholesterol content (1st row), saturation index (2nd row) double bond position and configuration (3rd row) and headgroup geometry and charge (4th row). Line corresponds to the median of individual biological replicates shown with different symbols (n≥3).

A

NR12A

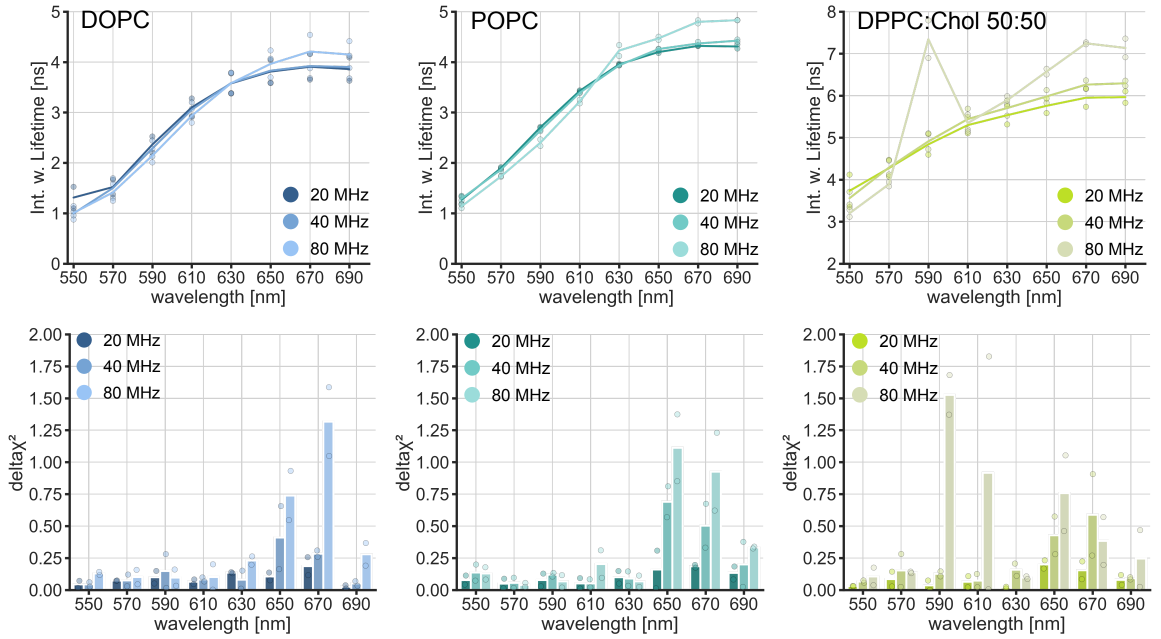

B

NR12S - DPPC:Chol 50:50

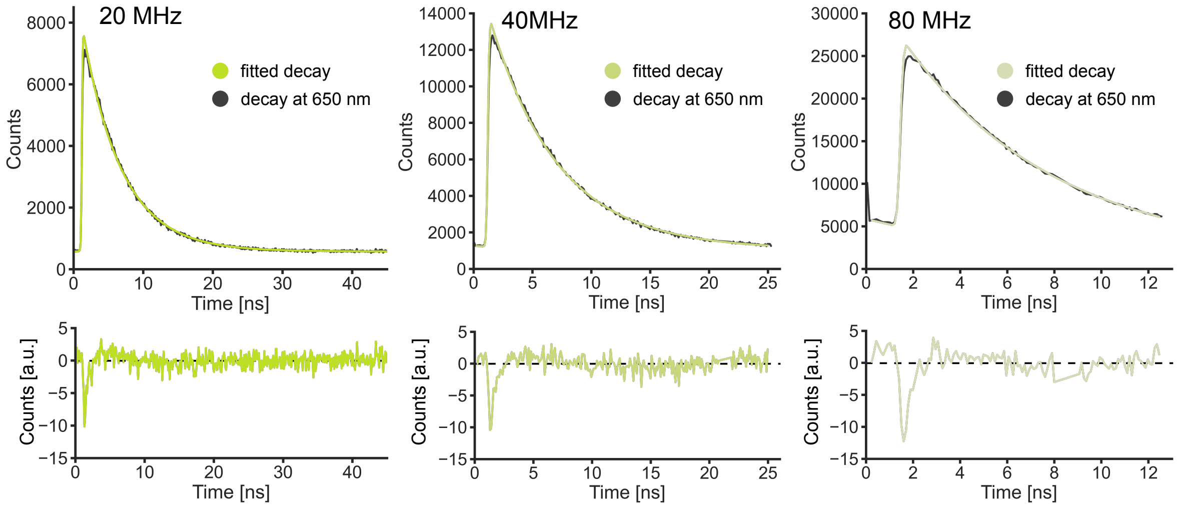

Figure S4: Influence of laser frequency on lifetime analysis. Spectral fluorescence lifetime measurements of the probes in LUVs were carried out within 500-700 nm in intervals of 20 nm. Multiexponential curve fitting was performed for the fluorescence decays (for details see Material and Methods). The estimated lifetimes of multiexponential curve fitting and the goodness of the fit are evaluated at different laser frequencies: 20, 40 and 80 MHz. A| Spectrally resolved intensity weighted lifetime of NR12A in in Δ9*cis* DOPC (left, blue), POPC (middle, cyan) and DPPC:Chol 50:50 (right, green) at different laser frequencies. The corresponding χ^2 values serve as indicator for the goodness of the fit and were obtained for each 20 nm interval and are shown below. The line and bar correspond to the median and average, respectively, of two technical replicates. B| Fluorescence decays (grey) and

their corresponding fit (green) of NR12S in DPPC:Chol 50:50 at 650 nm at different laser frequencies: 20 MHz (left), 40 MHz (middle) and 80 MHz (right). Corresponding residual counts in artificial units are shown below.

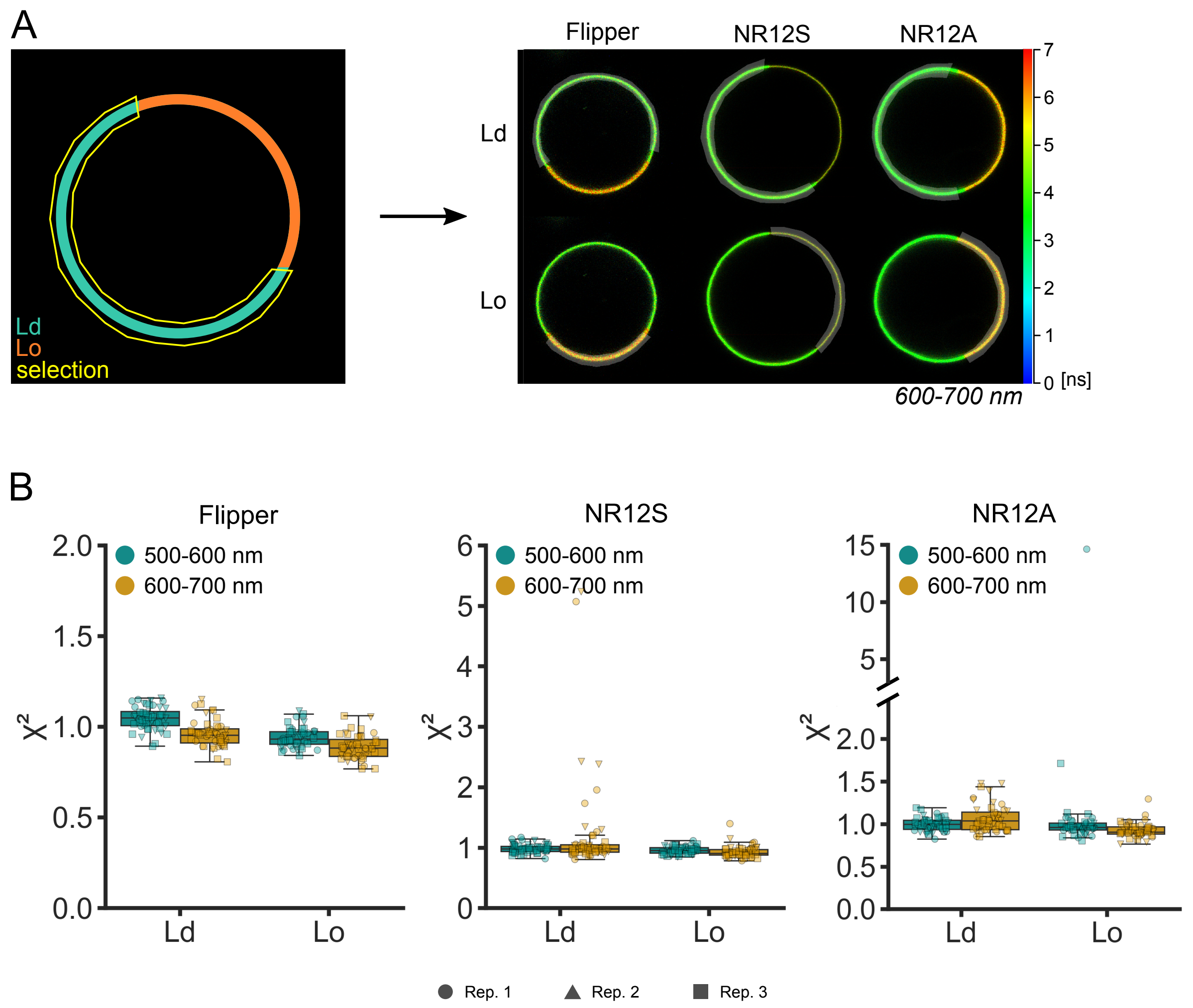

Figure S5: Phase selection and Chi-squared values of the multiexponential curve fitting of Flipper, NR12S and NR12A in phase-separated GUVs. Lifetime measurements in phase-separated GUVs were carried out at 500-600 nm or 600-700 nm emission. Multiexponential curve fitting was performed for the fluorescence decays (for details see Material and Methods). A| Overview of manual phase selection procedure in the LAS X software. Liquid disordered (Ld) and liquid-ordered (Lo) phase were selected separately for each phase-separated GUV and each selection was used for lifetime analysis at 500-600 nm and 600-700 nm. B| χ^2 values serve as indicator for the goodness of the fit and were obtained for Ld and Lo phase and are shown for Flipper (left), NR12S (middle) and NR12A (right) at 500-600 nm and 600-700 nm. Different symbols correspond to GUVs of individual biological replicates (n=3).

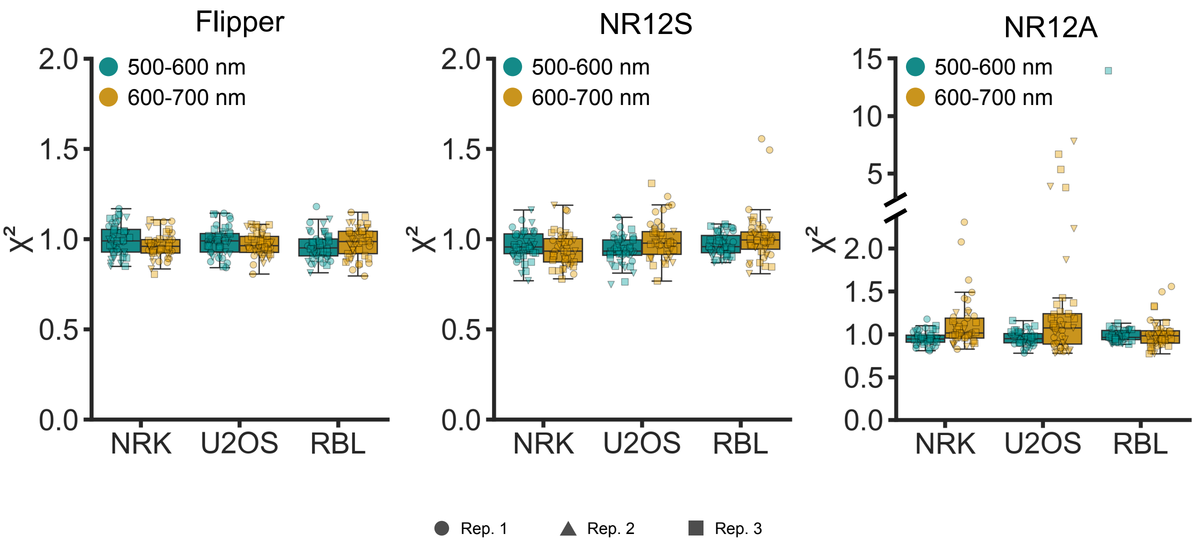

Figure S6: Chi-squared values of the multiexponential curve fitting of Flipper, NR12S and NR12A in different cell types. Lifetime measurements in NRK 52E, U2OS and RBL cells were carried out at 500-600 nm or 600-700 nm emission. Multiexponential curve fitting was performed for the whole-image fluorescence decays (for details see Material and Methods). χ^2 values serve as indicator for the goodness of the fit and were obtained for different cell types and are shown for Flipper (left), NR12S (middle) and NR12A (right) at 500-600 nm and 600-700 nm. Different symbols correspond to images of individual biological replicates (n=3).

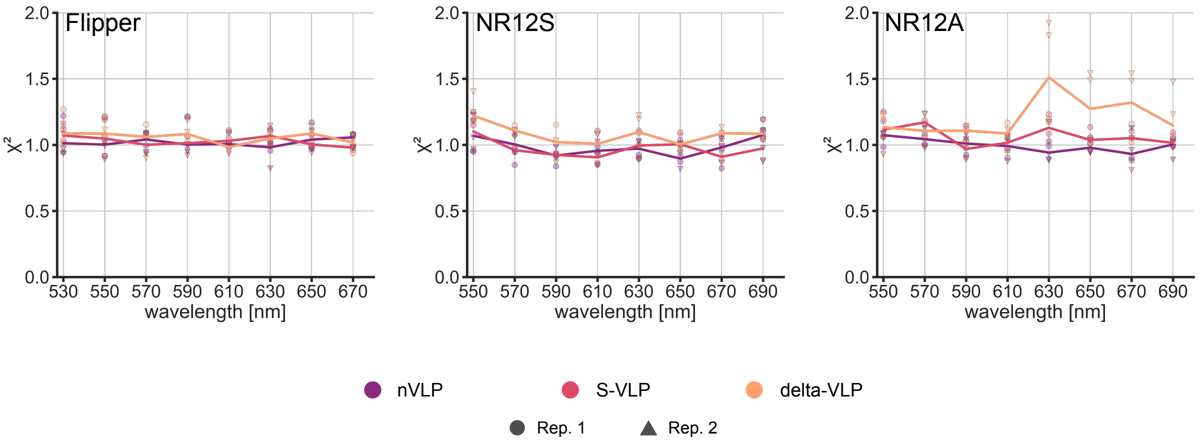

Figure S7: Chi-squared values of the multiexponential curve fitting of Flipper, NR12S and NR12A in different VLP species. Spectral fluorescence lifetime measurements of the probes in SARS-CoV-2 n-VLPs, S-VLPs or delta-VLPs were carried out within 500-700 nm in intervals of 20 nm. Multiexponential curve fitting was performed for the fluorescence decays (for details see Material and Methods). χ^2 values serve as indicator for the goodness of the fit and were obtained for each 20 nm interval and are shown for Flipper (left), NR12S (middle) and NR12A (right) in different VLP species. Line corresponds to the median of individual biological replicates shown with different symbols (n=2).
